# Diagnosis and Endoscopic Treatment of Hemobilia Due to Biliary Angiodysplasia: A Case Report and Literature Review

**DOI:** 10.7759/cureus.50552

**Published:** 2023-12-15

**Authors:** Marcus Vinícius S Costa, Lucas V Aragão, Julia M Jesus, Fabio C Mancini, Tomazo P Franzini

**Affiliations:** 1 Gastrointestinal Endoscopy, Hospital 9 de Julho, São Paulo, BRA; 2 Gastrointestial Endoscopy, Hospital 9 de Julho, São Paulo, BRA

**Keywords:** angiodysplasia, hemobilia, cholangioscopy, gastrointestinal bleeding, ercp

## Abstract

Hemobilia is described as bleeding from the intra- or extrahepatic biliary tree expressed through the major duodenal papilla into the duodenum, with angiodysplasia of the major biliary duct as a rare etiological factor with few cases reported in the literature. Cholangioscopy plays a pivotal role in diagnosing and making therapeutic decisions regarding biliary tract lesions. We report a case of the diagnosis and treatment of hemobilia secondary to bleeding from angiodysplasia of the major biliary duct, which was resolved after the placement of a fully covered metallic stent, with a review of the literature.

## Introduction

Hemobilia is an infrequent cause of upper gastrointestinal bleeding, which is most often secondary to intrabiliary procedures, hepatic trauma, inflammatory diseases, biliary tumors, and vascular malformations [1,2]. Although common in the gastrointestinal tract, angiodysplasia is extremely rare in the biliary ducts, with few cases described thus far. The resolution of these pathologies has become quicker and more precise with the advancement of cholangioscopy as a diagnostic and therapeutic tool [3]. Until now, the diagnosis of these conditions depended on radiological assistance and empirical treatments. We report a case of a patient with hemobilia due to angiodysplasia of the major biliary duct, diagnosed by cholangioscopy, and the condition was resolved after the placement of a biliary stent.

## Case presentation

An 87-year-old male patient with atrial fibrillation who was on apixaban 5 mg/day presented to the emergency department with exertional dyspnea and lower limb edema. He had no history of external bleeding but complained of mild abdominal pain upon examination. Laboratory tests revealed acute anemia with a hemoglobin level of 5.7 g/dL.

**Figure 1:**
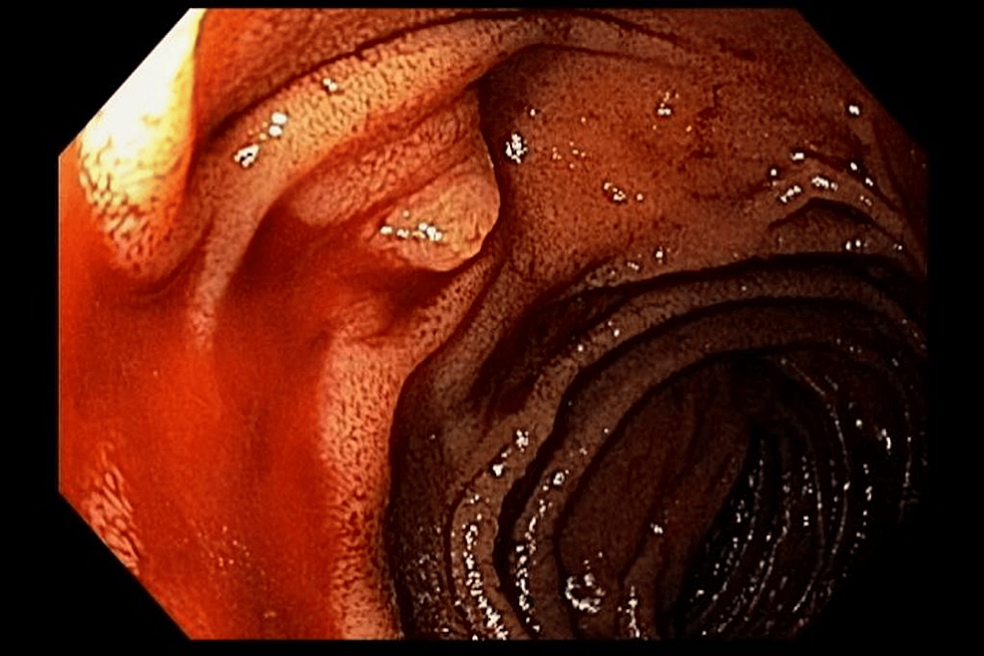
Active bleeding from the major duodenal papilla

Due to the patient's age and comorbidities, the attending team opted for a capsule endoscopy to avoid a more invasive procedure like a traditional endoscopy after an inconclusive computed tomography. Capsule endoscopy revealed blood residues in the stomach and small intestine, with recent clots predominantly in the duodenal region. Upper gastrointestinal endoscopy (EGD) identified active bleeding at the major duodenal papilla with an unsuccessful attempt at hemostasis through sclerosis (Figure 1).

**Figure 2:**
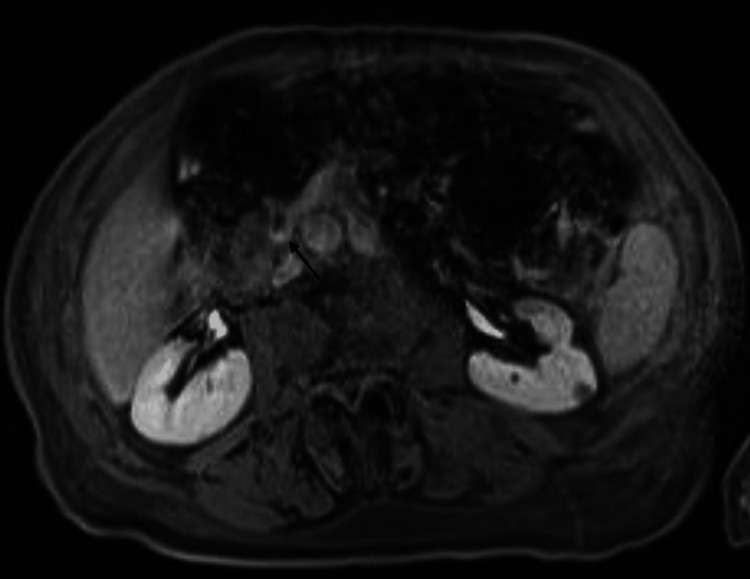
Enhancement focus in the distal common bile duct (black arrow)

Following an investigation with magnetic resonance imaging of the biliary tract, which identified a punctate enhancement focus in the distal common bile duct (Figure 2), the patient underwent endoscopic retrograde cholangiopancreatography (ERCP). During the procedure, bleeding at the papillary orifice persisted, and cholangiography showed no abnormalities.

**Figure 3:**
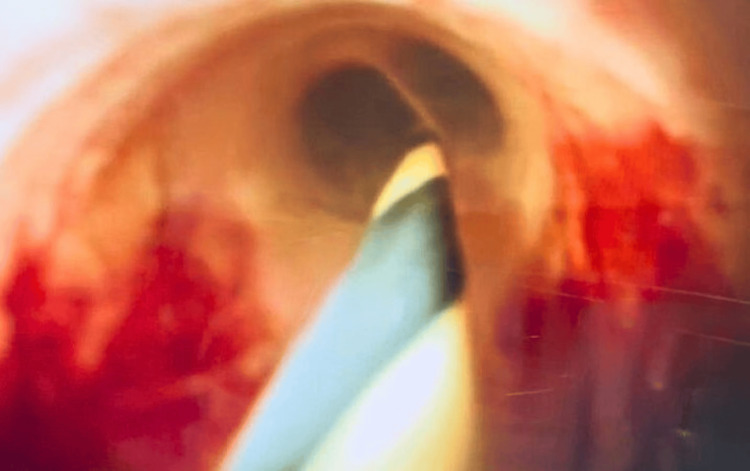
Transition between normal biliary mucosa and angiodysplasia on cholangioscopy

**Figure 4:**
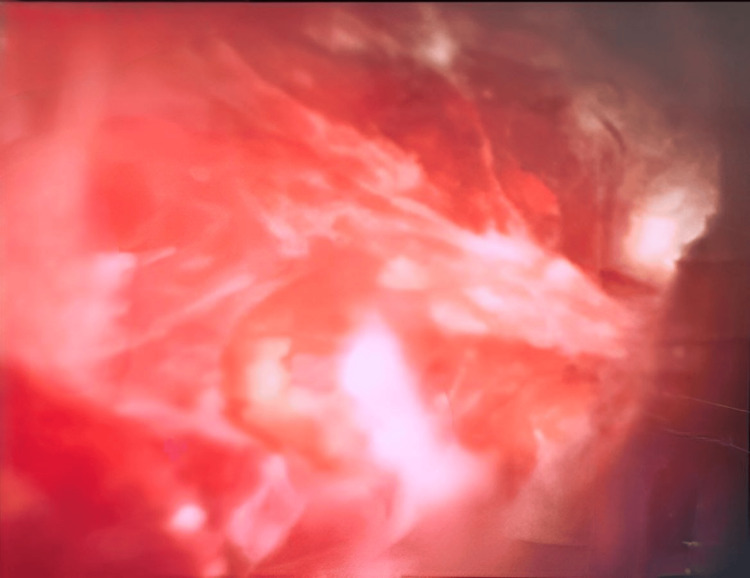
Abnormal vessels on cholangioscopy

Cholangioscopy with SpyScope DS® (Boston Scientific, Massachusetts) immediately above the papilla revealed anomalous vascular formations, suggestive of angiectasias with active bleeding (Figures 3, 4).

A 10 mm x 60 mm fully covered self-expanding metallic stent (WallFlex® Biliary RX Stent, Boston Scientific, Massachusetts) was placed for mechanical hemostasis, resulting in a favorable outcome.

The patient remained hospitalized, with stabilization of hematimetric values but developed acute cholecystitis due to obstruction of the cystic duct by the stent. He underwent laparoscopic cholecystectomy and was discharged on the fourth postoperative day. The stent was removed by EGD after 90 days, with resolution of the bleeding, and anticoagulation was resumed.

## Discussion

Hemobilia remains a challenging entity to diagnose and treat, requiring the use of various technologies and a multidisciplinary approach. Common causes include intrabiliary procedures (endoscopic or percutaneous), hepatic trauma, cholangiopathies secondary to cirrhosis, tumors, and vascular malformations. These, however, account for a minority of cases, usually occurring secondary to portobiliary fistulas, varices, hereditary telangiectasias, and rarely angiodysplasias [4,5].

Angiodysplasia is a common finding in upper and lower gastrointestinal endoscopies, primarily located in the stomach, duodenum, and colon. Its occurrence in the biliary tract is extremely rare, making diagnosis challenging and requiring specific resources and trained professionals, with few cases reported to date. It is characterized by the malformation of vessels in the mucosa and submucosa, with no hereditary or racial connection, but with a higher incidence in elderly patients with aortic stenosis, chronic kidney disease, lung diseases, and von Willebrand disease. Its pathophysiology remains uncertain but may be related to common bile duct contractions causing intermittent vessel obstruction, leading to focal dilations, tortuosities, and collateral vessel formation [6,7].

The advancement of endoscopic devices and technologies now allows for the determination of the causes of hemobilia. Once a diagnostic tool, ERCP has become an important therapeutic tool, evolving toward digital cholangioscopy. Adverse events, such as biliary tract perforation, air embolism, and bacteremia, are rare, though slightly higher than ERCP. Risk factors include patient age, stent placement, and, primarily, lithotripsy for biliary stones [8,9]. In our case, cholangioscopy played a crucial role in diagnosis and treatment. After an unsuccessful attempt at endoscopic treatment and cholangiography showing no major abnormalities, cholangioscopy revealed the source of bleeding as an angiodysplasia in the distal major biliary duct. The few cases reported in the literature have had varying approaches to this finding. One patient with a history of hemobilia, but no active bleeding at the time of diagnosis, maintained stable hemoglobin levels without intervention. Another used laser therapy for both angiodysplasia and actively bleeding neoplasms, and a third underwent radiointerventional embolization [3,10,11]. Factors influencing our treatment decision were active bleeding, a distal location near the papilla, and the unavailability of hemostasis accessories with SpyScope DS® at the time of the examination. Possible complications related to biliary stent placement include acute pancreatitis, cholangitis, migration, and acute cholecystitis. In patients eligible for laparoscopic cholecystectomy, the procedure can be performed as described in our case. Percutaneous or endoscopic ultrasound-guided gallbladder drainage is an alternative for gallbladder decompression in patients who are not surgical candidates [8,12].

## Conclusions

In conclusion, digital cholangioscopy played a crucial role in the diagnosis and treatment decision for hemobilia due to angiodysplasia of the major biliary duct. There is still a lack of data in the literature to define the gold-standard therapy, but the use of a fully covered metallic stent proved effective in achieving hemostasis.
